# Sex differences in skeletal muscle-aging trajectory: same processes, but with a different ranking

**DOI:** 10.1007/s11357-023-00750-4

**Published:** 2023-02-23

**Authors:** Jelle C.B.C. de Jong, Brecht J. Attema, Marjanne D. van der Hoek, Lars Verschuren, Martien P.M. Caspers, Robert Kleemann, Feike R. van der Leij, Anita M. van den Hoek, Arie G. Nieuwenhuizen, Jaap Keijer

**Affiliations:** 1 Human and Animal Physiology, Wageningen University, 6700AH, Wageningen, The Netherlands; 2 Department of Metabolic Health Research, The Netherlands Organization for Applied Scientific Research (TNO), Leiden, The Netherlands; 3 Applied Research Centre Food and Dairy, Van Hall Larenstein University of Applied Sciences, Leeuwarden, The Netherlands; 4 MCL Academy, Medical Centre Leeuwarden, Leeuwarden, The Netherlands; 5 Department of Microbiology and Systems Biology, The Netherlands Organization for Applied Scientific Research (TNO), Zeist, The Netherlands; 6 Research and Innovation Centre Agri, Food & Life Sciences, Inholland University of Applied Sciences, Delft and Amsterdam, The Netherlands

**Keywords:** Gender, Mitochondria, Oxidative phosphorylation, AKT signaling, Myofiber type, Frailty

## Abstract

Sex differences in muscle aging are poorly understood, but could be crucial for the optimization of sarcopenia-related interventions. To gain insight into potential sex differences in muscle aging, we recruited young (23 ± 2 years, 13 males and 13 females) and old (80 ± 3.5 years, 28 males and 26 females) participants. Males and females in both groups were highly matched, and *vastus lateralis* muscle parameters of old versus young participants were compared for each sex separately, focusing on gene expression. The overall gene expression profiles separated the sexes, but similar gene expression patterns separated old from young participants in males and females. Genes were indeed regulated in the same direction in both sexes during aging; however, the magnitude of differential expression was sex specific. In males, oxidative phosphorylation was the top-ranked differentially expressed process, and in females, this was cell growth mediated by AKT signaling. Findings from RNA-seq data were studied in greater detail using alternative approaches. In addition, we confirmed our data using publicly available data from three independent human studies. In conclusion, top-ranked pathways differ between males and females, but were present and altered in the same direction in both sexes. We conclude that the same processes are associated with skeletal muscle aging in males and females, but the differential expression of those processes in old vs. young participants is sex specific.

**Supplementary Information:**

The online version contains supplementary material available at 10.1007/s11357-023-00750-4.

## Introduction

Sarcopenia is an aging-related muscle disease characterized by loss of muscle strength and mass [1, 2]. Loss of muscle in the elderly increases the risk for, among others, falls and fractures [3], physical dependence [4], and hospitalization [5], and is associated with comorbidities such as diabetes mellitus type 2 and cardiovascular disease [6–9]. This makes sarcopenia a critical driver of frailty and mortality in the elderly [10, 11], with a concomitant impact on healthcare costs [12, 13]. It is a major public health issue worldwide, with its prevalence expected to increase during the next few decades [14]. It is therefore essential to increase our understanding of the pathophysiology of muscle aging, to open up perspectives on novel therapeutic interventions to prevent or reverse sarcopenia.

The etiology underlying sarcopenia is complex and could be sex specific, as sex differences have been reported in the prevalence and functional manifestation of sarcopenia. For example, higher rates of loss of muscle mass during aging have been reported in males compared to females [15]. In addition, a higher prevalence in the rate of sarcopenia in males compared to females has been reported [16], although this could be dependent on the methods of diagnosis being used [17]. Such sex differences in the prevalence and manifestation of sarcopenia could result from (partial) sex-dependent mechanisms underlying sarcopenia. Indeed, some studies have reported sex-specific markers for sarcopenia; however, the number of studies on this topic is limited [18–21].

Well-studied drivers of age-related loss of muscle mass and strength include a decreased mitochondrial content or function [22, 23]. One electron microscopy study measured mitochondrial content and found that intermyofibrillar mitochondrial size was primarily decreased in old females and not in old males [18]. Moreover, in the FITAAL study, we previously found that intramuscular (acetyl) carnitine levels decreased with aging in females, but not in males [19]. Together, these two studies suggest that during aging, females are exposed to more changes in mitochondrial content and function compared to males. Furthermore, the composition of the plasma proteome is also known to change during aging, and interestingly, a large human study found that these age-associated changes were highly sex specific [20]. Another study showed that higher serum myostatin levels were associated with sarcopenia in males, while in females, lower serum IGF1 levels were associated with sarcopenia [21]. Together, these studies suggest that a variety of biological processes associated with aging could be sex specific.

These findings motivate us to study sex differences in human muscle aging, as this could lead to the development of sex-specific or stratified interventions for sarcopenia. Since sex differences in a variety of biological processes were reported, our aim was to investigate how sex differences are manifested in human muscle tissue from old and young males and females on a molecular level. To do so, we used *vastus lateralis* muscle from participants of the FITAAL study, a cross-sectional study with homogenous, highly matched young and old (>75 years of age) male and female participant groups [19]. Gene expression was analyzed, and the most notable findings were confirmed using alternative methods and approaches. Lastly, we verified these findings using publicly available data from three independent human studies with comparable study populations.

## Materials and methods

### Study design, population, and ethics statement

Functional data, muscle biopsies, and serum samples were obtained during the FITAAL study [19]. Briefly, the FITAAL study is a cross-sectional human study in which 13 male and 13 female young individuals (20–30 years of age) and 26 elderly males and 28 elderly females (75+ years of age) participated. Male and female participants were highly matched in both young and old groups with respect to age and BMI. Males and females were also matched for the Fried frailty score in the old groups. Exclusion criteria included diagnosis with cardiac failure, COPD, anemia, cancer, neuromuscular disorder or dementia, contraindication for muscle biopsy, recent (up to 3 months prior to initiation of the study) significant medical or surgical events or treatment by a medical specialist, current enrolment in another study, intake of carnitine supplements, or usage of several types of medication (e.g., corticosteroids or fibrates). An abnormal BMI (<20 kg/m^2^ or >25 kg/m^2^), diagnosis with diabetes mellitus type I and II, and a high frequency of physical exercise (>4 times a week) served as additional exclusion criteria for young individuals, as did pregnancy or nursing for young female participants. The study was conducted according to the declaration of Helsinki, was approved by the medical ethical committee of Wageningen University (METC nr. 16/20), and is registered in the Dutch Trial Register (NTR6124). All participants provided written informed consent prior to enrolment. An overview of subject characteristics can be found in Table 1.

**Table 1:** Participant characteristics

	Males		Females	
	Young (*n* = 13)	Old (*n* = 28)	Young (*n* = 13)	Old (*n* = 26)
Age (years)	23.3 ± 1.9^a^	79.7 ± 3.5^b^	22.6 ± 1.9^a^	80.2 ± 3.1^b^
Weight (kg)	76.2 ± 8.7^a^	81.0 ± 11.1^a^	63.9 ± 5.8^b^	68.9 ± 10.3^b^
BMI (kg/m^2^)	22.5 ± 1.1^a^	26.4 ± 3.9^b^	22.2 ± 1.7^a^	26.2 ± 3.2^b^
Body fat (%)	16.9 ± 3.3^a^	25.3 ± 4.6^b^	29.1 ± 3.9^c^	35.7 ± 4.1^d^
Lean mass (%)	79.3 ± 3.2^a^	71.3 ± 4.3^b^	67.3 ± 3.8^c^	61.4 ± 4.0^d^
BMC (%)	3.7 ± 0.3^a^	3.3 ± 0.5^b^	3.6 ± 0.4^a^	2.9 ± 0.5^b^
Handgrip strength (kg)	-	32.0 ± 9.5^a^	-	22.2 ± 5.9^b^
400m walk test (s)	-	323.3 ± 40.1^a^	-	393.9 ± 158.2^b^
SPPB (points)	-	10.0 ± 1.9^a^	-	8.9 ± 3.0^a^
Fried frailty score	-	0.5 ± 0.6^a^	-	0.7 ± 1.0^a^

The presence of different letters (a–d) across a row indicates significant differences among respective groups. Values are averages ± SD

### Body composition and muscle biopsy

Body weight and height were measured to the nearest 0.1 kg or 0.1 cm, respectively; 1 kg was subtracted to correct for clothing. Body composition was measured using a DEXA scan (Hologic Discovery-A, Hologic Inc., Bedford, USA). Muscle tissue samples were collected by a trained physician at Leeuwarden Medical Centre, and a percutaneous needle biopsy was taken (50–80 mg) from the *vastus lateralis* muscle, according to the Bergström method with suction [24, 25]. Samples were taken after an overnight fast, under local anesthesia and taken at the thickest part of the muscle, approximately 15 to 20 cm above the edge of the patella. Samples for RNA extraction were immediately snap-frozen by immersion in liquid nitrogen. Samples for immunohistochemistry were snap frozen in isopentane cooled in a liquid nitrogen bath. All muscle biopsy samples were subsequently stored at −80 °C prior to further analyses.

### RNA extraction and gene expression profiling

Total RNA was extracted from the muscle biopsies using an RNA isolation kit with NucleoSpin columns (kit#740955, Macherey-Nagel). Total RNA concentration was determined spectrophotometrically using Nanodrop 1000 (Isogen Life Science, De Meern, the Netherlands), and RNA quality was assessed using the 2100 Bioanalyzer (Agilent Technologies, Amstelveen, the Netherlands). The NEBNext Ultra Directional RNA Library Prep Kit for Illumina was used to process the samples according to the protocol “NEBNext Ultra Directional RNA Library Prep Kit for Illumina” (NEB #E7420S/L). Strand-specific messenger RNA sequencing libraries were generated and sequenced at GenomeScan (Leiden, the Netherlands). The libraries were multiplexed, clustered, and sequenced on an Illumina NextSeq500 with a single-read 75-cycle sequencing protocol, 15 million reads per sample. Sequence reads were quality trimmed and mapped to the reference genome *Homo sapiens* GRCh38 using the trimmomatic and STAR-aligner software (GitHub). Read counts per gene transcript (counts/feature) were obtained from htseq-count software (GitHub).

### Immunohistochemistry

Sections of 7 μm were cut using a cryostat with a temperature set to −20 °C, and sections were stored at −80 °C until analysis. Sections were air-dried for 30 min and fixated in 4% paraformaldehyde for 15 min. Antigen retrieval was performed by incubating the sections in a sodium-citrate buffer (10mM, pH 6) that was kept at sub-boiling temperature for 15 min using microwave heating. Free aldehyde groups were masked by a 20-min incubation in 1.5% glycine, and blocking was performed by means of a 30-min incubation in 5% normal goat serum. After blocking, slides were incubated with primary antibodies for MYH7 (1:200, SAB4200670, Sigma-Aldrich) and dystrophin (1:100, ab85302, Abcam) diluted in 0.05% acetylated bovine serum (900.099, Aurion) and kept at room temperature for 1 h. Other slides were incubated with primary antibodies for COX4 (1:200, ab16056, Abcam) or a mixture of p-AKT^(thr308)^ (1:200, 13038S, Cell Signaling) and AKT (1:200, 2920S, Cell Signaling) diluted in 0.05% acetylated bovine serum albumin and kept overnight at 4 °C. For negative controls, the incubation step with the primary antibody was omitted. Next, slides were incubated with goat anti-rabbit IgG Alexa Fluor 488 secondary antibody (1:1000, A-11008, ThermoFisher) or goat anti-rabbit IgG Alexa Fluor 594 secondary antibody (1:1000, A-11012, ThermoFisher). Finally, slides were counterstained using DAPI, and slides were covered using Fluoromount-g (0100-01, Southernbiotech).

Tile scans of whole sections were made at 20× magnification using a fluorescence microscope (Leica DM6B) and a digital camera (DFC365 FX). Using ImageJ, slow myofibers were identified by separating the color channels and selecting the green channel (containing immunolabeled MYH7, a slow myofiber-specific marker). Thresholding was applied to select the area of the slow myofibers, and myofiber size was measured using the “analyze particle” function in ImageJ. The minimal Ferret’s diameter was used as the read-out parameter since this parameter was reported to be the least sensitive for the sectioning angle [26]. Fast myofibers were identified by thresholding the red channel (containing immunolabeled dystrophin) and subtracting the area of the green channel (slow myofibers). This provides a picture containing the area of fast myofibers only, which was used to measure the minimal Ferret’s diameter. The number of myofiber types was counted manually using whole-section pictures by a blinded investigator. Biopsies with less than 70 myofibers were excluded [27]. For intensity measurements, whole-section scans at 20× magnification were made using at least 6-step *z*-stacks, and LAS-X software was used for maximum projection processing. Whole-section pictures were exported, and signal intensity was measured automatically using ImageJ. The thresholding function was used to select tissue area, and the average intensity was measured (settings were kept constant throughout all measurements).

### Serum collection and IGF1 serum ELISA

Blood samples were taken from participants in the morning after an overnight fast and collected using serum-separating tubes (BD diagnostics). Serum was stored at −80 °C until analysis. IGF1 concentrations were determined using a commercial ELISA kit (DG100B, R&D Systems). Assays were performed according to the manufacturer’s instructions. The average intra-assay coefficient of variation was 5.8 ± 4.6%, and the average inter-assay coefficient of variation was 8.9 ± 2.0%.

### Transcriptome data analysis

RNA-seq-derived count data was normalized, and log2fold change values of old vs. young groups were calculated using the DESeq2 package in R [28]. Genes were considered significantly differentially expressed if *p*-values were lower than 0.01. For pathway analysis, DEGs were used as input for Ingenuity Pathway Analysis (www.ingenuity.com, accessed 2021). For principal component analysis, normalized counts were used as input using MetaboAnalyst 5.0 (www.metaboanalyst.ca, accessed 2021). The gene expression dataset can be accessed from the Gene Expression Omnibus (GEO) with accession number GSE144304.

Furthermore, to compare our findings to those of other studies, 46 unique GEO datasets were found using the search term (*human ageing “vastus lateralis*”), and eventually, 3 datasets were included in our analysis [29–32]. Other GEO datasets were not used for various reasons; e.g., due to the absence of female participants, muscle biopsies were taken only after an intervention or from another muscle than the *vastus lateralis* muscle (see Supplementary Fig. 1 for an overview). GSE8479, GSE362&674, and GSE157585 were downloaded from GEO (Table 2). Gene expression profiles of old versus young participants were compared. In the case of intervention studies, profiles of baseline measurements were used. If for one gene multiple probes were used, the average -log(*p*-value) and log2FC of all significantly differentially expressed probes was calculated and used as the value for the gene. If no probe of a gene was significantly differentially expressed, then the average -log(*p*-value) and log2FC of all probes was calculated.

**Table 2:** Overview of included GEO datasets

Study	Number of participants (m/f)		Age range (years: min–max)		
	Young	Old	Young	Old	Biopted muscle
This study (GSE144304)	26 (13/13)	54 (28/26)	20–27	75–90	*Vastus lateralis*
GSE157585 [29]	21 (10/11)	30 (16/16)	20–30	65–91	*Vastus lateralis*
GSE8479 [30]	26 (12/14)	25 (13/12)	18–28	65–84	*Vastus lateralis*
GSE362 and GSE674 [31, 32]	14 (7/7)	16 (8/8)	20–29	65–75	*Vastus lateralis*
Total	**87**	**125**	**18-30**	**≥65**	

Total number of participants across the included studies and age ranges are provided in bold

### Data analysis

Statistical differences (of data other than RNA-seq data) between old vs. young groups were determined using an unpaired *t*-test. If data was not normally distributed, then a Mann-Whitney *U* test was used. If statistical differences between multiple groups were tested, then a two-way ANOVA, followed by a Tukey’s test for post-hoc analysis, was used. If data was not normally distributed, then a Kruskal-Wallis test, followed by a Mann-Whitney U test, was used. The normality of data was tested using D’Agostino and Pearson omnibus normality test. Correlations between two variables were tested for significance using regression analysis. A *p*-value of <0.05 was considered statistically significant, and all values are displayed as mean ± SEM unless mentioned otherwise. Two-tailed *p*-values were used, and these analyses were performed using GraphPad Prism version 5.04 (GraphPad Software, CA, USA) or IBM SPSS Statistics 25 (IBM, NY, USA).

## Results

### Males lose more lean mass than females in their arms, but not in their legs

**Fig. 1:**
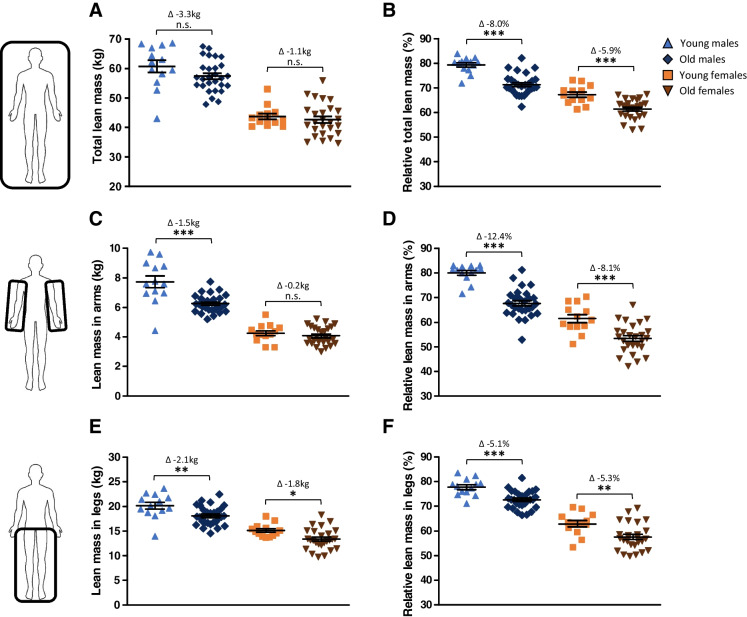
Lean mass data measured using DEXA scans. Data of young males (*n* = 13), old males (*n* = 28), young females (*n* = 13), and old females (*n* = 26) are shown. (**A**) Absolute total lean body mass. (**B**) Relative total lean body mass. (**C**) Absolute lean mass in arms only. (**D**) Relative lean mass in arms only. (**E**) Absolute lean mass in legs only. (**F**) Relative lean mass in legs only. Values represent mean ± SEM, *p < 0.05, **p < 0.01, and ***p < 0.001

The mean age and BMI of male and female participants was not different in both young and old groups. The Fried frailty score of old male and female participants did also not differ between the two sexes (Table 1). Loss of total absolute lean mass was not detected in either sex (Fig. 1A), but relative total lean mass was significantly lower in old vs. young participants of both sexes (both *p* < 0.001, Fig. 1B). In the arms, old males had 1.5 kg less lean mass compared to young males (*p* < 0.001, Fig. 1C), while old females did not have less lean mass in their arms compared to young females (*p* = 0.38, Fig. 1C). The relative lean mass of the arms was lower in old compared to young participants of both sexes (both *p* < 0.001, Fig. 1D). In the legs, a lower absolute lean mass was observed in both old males (−2.1 kg, *p* < 0.01) and old females (−1.8 kg, *p* < 0.05) compared to the young groups (Fig. 1E). The relative lean mass of the legs was also lower in the old compared to the young groups of either sex (Fig. 1F). These results suggest that the interaction of sex with muscle aging varies between muscle groups and that results from one muscle group may not necessarily be extrapolated to other muscle groups of the body.

### Aging has sex-specific effects on the proportion of slow myofibers, but not on myofiber diameter

**Fig. 2:**
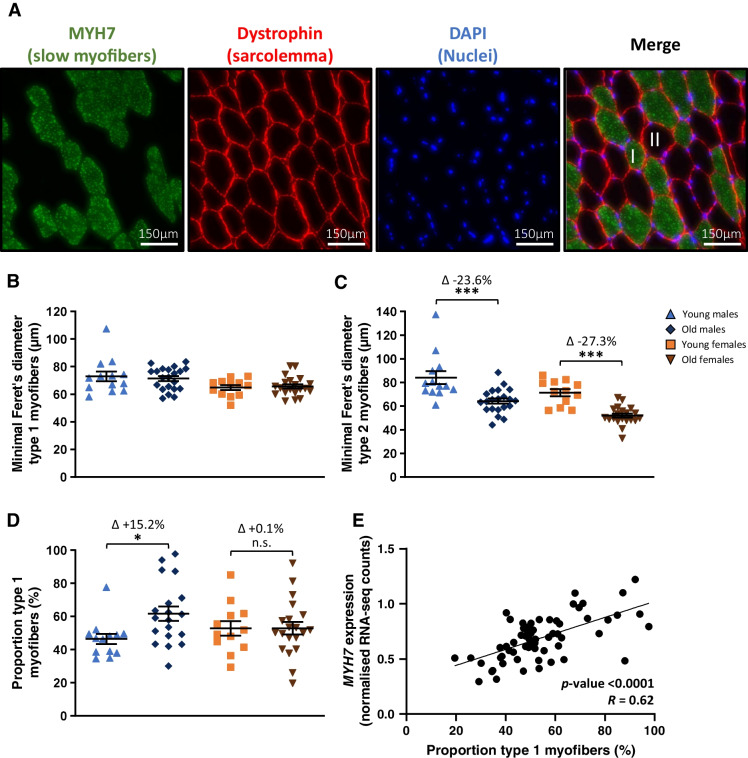
Myofiber typing and diameter of *vastus lateralis* muscle*.* Data of young males (*n* = 13), old males (*n* = 21), young females (*n* = 12), and old females (*n* = 20) are shown. (**A**) Representative pictures of immunofluorescence staining used to discriminate type 1 and type 2 myofibers (20× magnification). Pictures from left to right show staining of Myosin Heavy Chain 7 (type 1 myofibers, green), Dystrophin (sarcolemma, red), nuclei (DAPI in blue), and the merge of all channels. (**B**) Minimum Feret’s diameter of type 1 and (**C**) type 2 myofibers. (**D**) Percentage of type 1 and 2 myofibers. (**E**) Correlation between *MYH7* expression (RNA-seq derived data) and proportion of type 1 myofibers. Values represent mean ± SEM, *p < 0.05, **p < 0.01, and ***p < 0.001

To obtain insight into possible structural changes in the skeletal muscles, immunofluorescence staining of *vastus lateralis* muscle was used to identify type 1 (slow) and type 2 (fast) myofibers (Fig. 2A). The diameter of type 1 myofibers was not different in old compared to young participants of both sexes (Fig. 2B). A smaller diameter of type 2 myofibers was observed in old males (64.2 μm ± 2.2) compared to young males (84.0 μm ± 5.5, *p* < 0.001) and in old females (51.8 μm ± 1.7) compared to young females (71.4 μm ± 3.0, *p* < 0.001, Fig. 2C). Frequency graphs of the size of type 1 and 2 myofibers are provided as Supplementary Fig. 2.

The proportion of type 1 myofibers was higher in old males (61.6% ± 4.3) compared to young males (46.4% ± 3.1, *p* < 0.01), while no difference in the proportion of type 1 myofibers was found between old females (52.9% ± 3.8) compared to young females (52.8% ± 4.3, Fig. 2D). RNA-seq-based gene expression of Myosin Heavy Chain 7 (*MYH7*), a type 1 myofiber marker, significantly correlated with the observed percentage of type 1 myofibers (*R* = 0.62, *p* < 0.001, Fig. 2E), which cross-validated both techniques.

### Transcriptome profiles of males and females are different, but aging trajectory is largely shared

**Fig. 3:**
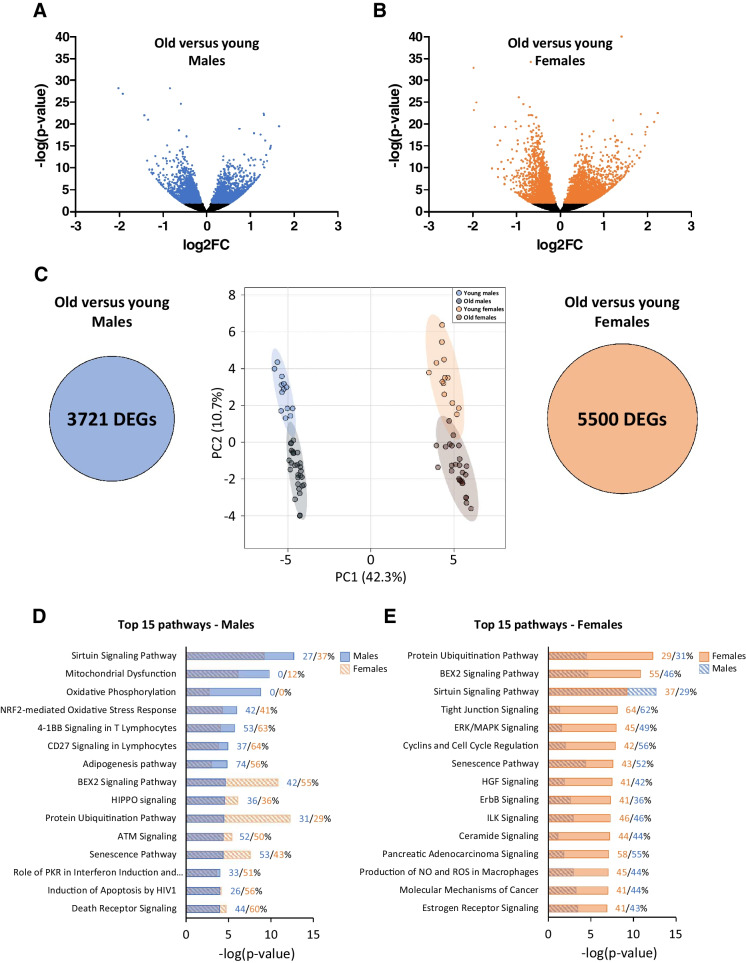
Overview of *vastus lateralis* RNA-seq data. Volcano plot of (**A**) old vs. young males (*n* = 28 and *n* = 13, respectively) and (**B**) old vs. young females (*n* = 26 and *n* = 13, respectively). (**C**) Venn diagrams of the total amount of DEGs in old vs. young males (left) and females (right). Principal component analysis plot based on top 5000 regulated genes across all groups and circles around individual data points represent 95% confidence regions per group (middle). (**D**) Top 15 differentially expressed pathways of old vs. young males. In blue bars are the -log(*p*-values) of males overlaying the -log(*p*-values) bars of the same pathways in females (orange with diagonal stripes). Percentages behind bars indicate the percentage of upregulated DEGs in the respective pathway. (**E**) Top 15 differentially expressed pathways of old vs. young females. In orange bars are the -log(*p*-values) of females overlaying the -log(*p*-values) bars of the same pathways of males (blue with diagonal stripes). Percentages behind bars indicate the percentage of upregulated DEGs in the respective pathway. Genes with -log(*p*-value) > 2 were considered to be differentially expressed

Analysis of RNA sequencing-based gene expression of *vastus lateralis* muscle biopsies revealed that 1932 differentially expressed genes (DEGs) were upregulated and 1789 DEGs were downregulated in old vs. young males (Fig. 3A), while in old vs. young females, 3117 DEGs were upregulated and 2383 DEGs were downregulated (Fig. 3B). In total, 3721 genes were differentially expressed in old vs. young males (Fig. 3C, left), while more genes (5500) were differentially expressed in old vs. young females (Fig. 3C, right). Principal component analysis revealed a clear separation of all four groups, suggesting that each group had a distinct gene expression pattern (Fig. 3C, middle). The first principal component explained a major part of the variation within the data, namely 42.3%, and separated males from females in both young and old groups. This shows that sex differences in the muscular transcriptome were present in both old and young groups and were largely preserved during aging. The second principal component, explaining 10.7% of the variation, separated the old from the young participants of each sex. Interestingly, the young and old groups of males and females were orientated in a parallel manner, which suggested that aging-related changes in gene expression are largely shared by the two sexes.

To gain insight into the biological processes that were represented by these genes, DEGs were used as input for pathway analysis for each sex separately because of the clear sex-related separation in the principal component analysis. The top 15 differentially expressed pathways in males included pathways related to (mitochondrial) metabolism, oxidative stress, inflammation and cell growth, and apoptosis signaling (Fig. 3D). Noticeably, for pathways related to (mitochondrial) metabolism (sirtuin signaling pathway, mitochondrial dysfunction and oxidative phosphorylation), males consistently had a higher -log(*p*-value) compared to females, and genes in these pathways were mostly downregulated in both sexes, as indicated by the percentage of upregulated genes in Fig. 3D. The top 15 differentially expressed pathways in females included pathways related to protein breakdown, growth signaling, inflammation, and estrogen receptor signaling (Fig. 3E). Noticeably, some of these pathways were significantly differentially expressed in females only, and the percentage of DEGs that were upregulated was largely the same in both sexes in these pathways. Furthermore, upstream regulator analysis was performed, and the top predicted upstream regulators are attached as Supplementary Fig. 3. To further investigate gene expression patterns that were unique for one of the sexes, we performed additional pathway analyses for DEGs that were unique or shared for the sexes.

### Sex-specific DEGs are largely regulated in the same direction by both sexes

**Fig. 4:**
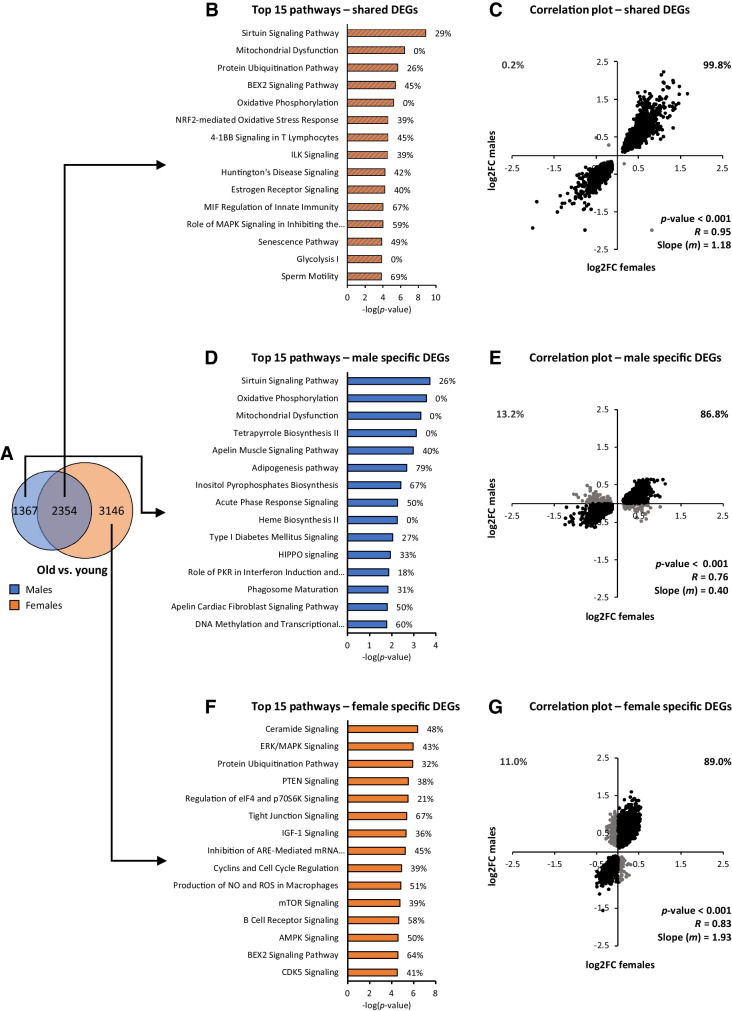
Pathway analyses and correlation plots of shared or sex-specific DEGs. (**A**) Venn diagram of the number of DEGs of old vs. young participants that are regulated in both sexes, or in only one of the sexes. (**B**) Top 15 differentially expressed pathways based on shared DEGs only. (**C**) Correlation plot of fold change values of all shared DEGs. (**D**) Top 15 differentially expressed pathways based on male-specific DEGs only. (**E**) Correlation plot of fold change values of male-specific DEGs only. (**F**) Top 15 differentially expressed pathways based on female-specific DEGs only. (**G**) Correlation plot of fold change values of female-specific DEGs only. In all correlation plots, *p*, *R*, and *m* values are based on all data points in a plot. Fold change values of old vs. young males are plotted on the *x*-axis, and fold change values of old vs. young females are plotted on the *y*-axis. Percentages in the upper corners of correlation plots represent the percentage of DEGs regulated in the opposite (Q1 and Q4) or same (Q2 and Q3) direction in males and females. Percentages behind bars (in panels B, D, and F) indicate the percentage of upregulated DEGs in the respective pathway

A Venn diagram of both male and female DEGs revealed that a part of the male and female DEGs was shared between the sexes, but also that a minority of the male DEGs were unique for the males, and a majority of the female DEGs were unique for the females (Fig. 4A). Using the 2354 shared DEGs as input for pathway analysis, top 15 regulated pathways included pathways related to (mitochondrial) metabolism, oxidative stress, inflammation, cellular growth, or protein breakdown (Fig. 4B). Importantly, when we tested the similarity of direction of regulation (up- or downregulation) using a correlation plot of the log fold change (log2FC) values of all 2354 shared DEGs, a strong correlation (*p* < 0.001, *R* = 0.95) was revealed (Fig. 4C). The direction of regulation was the same in 99.8% of the genes, and their fold change values were highly similar as well, as indicated by the slope-value *m* being almost 1, namely 1.18 (Fig. 4C).

In males, 1367 male-specific DEGs were found (Fig. 4A). Using these male-specific DEGs as input for pathway analysis, we found pathways related to (mitochondrial) metabolism, heme biosynthesis, apelin signaling, cell proliferation, and inflammation (Fig. 4D). After performing a correlation analysis for these male-specific DEGs, a significant correlation was revealed (*p* < 0.001, *R* = 0.76) between the log2FC values of males and females (Fig. 4E). Notably, in females, 86.8% of the male-specific DEGs were regulated in the same direction; however, the fold change values were higher in males compared to females as indicated by the slope-value *m* of 0.40.

In females, 3146 female-specific DEGs were found (Fig. 4A). Using these female-specific DEGs as input for pathway analysis, the top 15 regulated pathways included pathways related to growth signaling, protein breakdown, inflammation, and neural development (Fig. 4F). Log2FC values of female-specific DEGs correlated significantly between the two sexes (*p* < 0.001, *R* = 0.79, Fig. 4G). In males, 89% of the female-specific DEGs were regulated in the same direction, indicating that the direction of regulation was again highly similar between the sexes, as was found for the male-specific DEGs as well. Fold change values were higher in females compared to males, as indicated by the slope-value *m* of 1.93.

Our results indicated that aging-related changes in gene expression are primarily sex specific due to differences in the magnitude of these changes, but not due to differences in the direction of regulation (up or down). This suggests that the nature of sex-specific differences in the aging trajectory of skeletal muscle can primarily be found in the magnitude of differential expression since the majority of the sex-specific DEGs did differ in *p*-value, but did not differ with regards to the direction of regulation. To investigate this notion further, we performed additional in-depth analyses of the most prominent differentially expressed pathways of the male- and female-specific DEGs.

### Aging-associated loss of OXPHOS subunits is not sex specific in vastus lateralis muscle

**Fig. 5:**
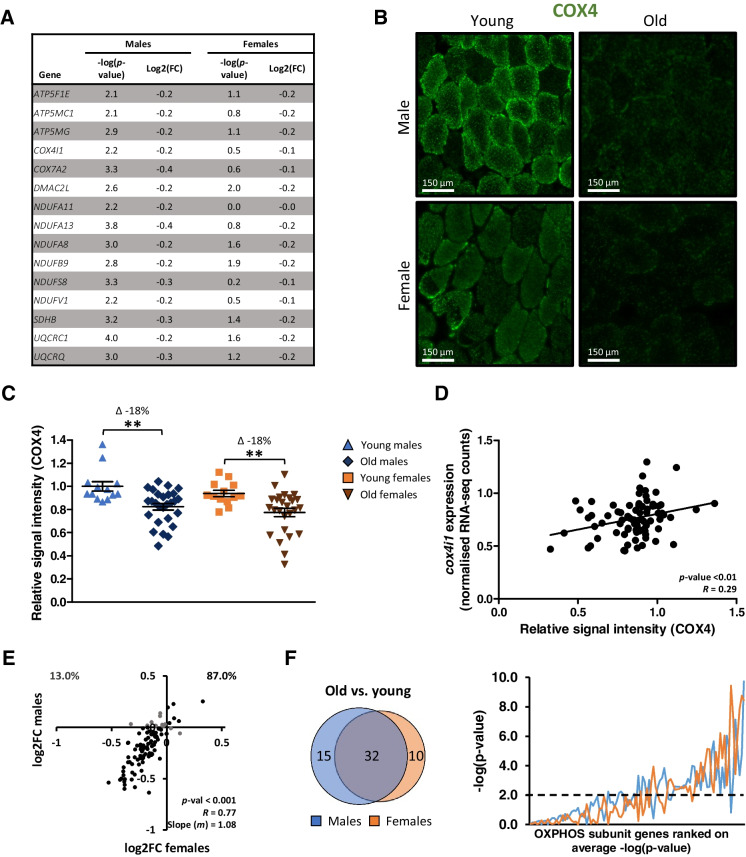
Effects of aging on OXPHOS subunits in males and females. (**A**) Table of all genes encoding for OXPHOS subunits (from MitoCarta 3.0 inventory) that were significantly regulated in males, but not in females. (**B**) Representative pictures of COX4 immunofluorescence staining (20× magnification). (**C**) Relative COX4 protein content in young males (*n* = 13), old males (*n* = 27), young females (*n* = 13), and old females (*n* = 26). (**D**) Correlation plot of the relative protein content of COX4 and normalized RNA-seq counts of *cox4i1* of all participants (*n* = 79). (**E**) Correlation plot of log2FC values of genes encoding for OXPHOS subunits, with fold change values of old vs. young males on the *x*-axis and old vs. young females on the *y*-axis. *P*, *R*, and *m* values are based on all data points in this plot. Percentages in the upper corners of this correlation plot represent the percentage of DEGs regulated in the opposite (Q1 and Q4) or the same (Q2 and Q3) directions in males and females. (**F**) Venn diagram of the number of genes encoding for OXPHOS subunits that are regulated in both or only one of the sexes (left). Line graph of *p*-values of OXPHOS subunit genes ranked on their average -log(*p*-value) in males and females (right)

The top three differentially expressed pathways of apparent male-specific DEGs were all related to oxidative phosphorylation (OXPHOS). Therefore, we chose to select and investigate male-specific DEGs encoding for mitochondrial subunits, using the MitoCarta 3.0 inventory [33], based on an age-dependent significant differential expression in males and not females. Fifteen male-specific DEGs were found (Fig. 5A). Close inspection revealed that, similar to the male regulation, all of these genes also displayed a negative log2FC value in females. Among the male-“specific” DEGs was *COX4I1*, which we decided to analyze at the protein level using immunohistochemistry of embedded tissue (Fig. 5B). Signal intensity of COX4 staining was significantly lower in old males compared to young males (−18%, *p* < 0.01), but also in old females compared to young females (−18%, *p* < 0.01, Fig. 5C). In addition, the signal intensity of COX4 staining correlated significantly with normalized RNA-seq *COX4I1* counts (*p* = 0.01, *R* = 0.29, Fig. 5D).

Based on these observations, loss of (some) OXPHOS subunits seemed to be more prominent in men, but did not seem to be sex specific. In addition, a significant correlation of the log2FC values of all genes encoding mitochondrial subunits in males and females was found (*p* < 0.001, *R* = 0.77, slope-value *m* = 1.08), indicating that also in the direction of regulation and fold change values, no sex effects were observed (Fig. 5E). Furthermore, the majority of the DEGs encoding for mitochondrial OXPHOS subunits were differentially expressed in both sexes (32 out of 57), 15 DEGs were differentially expressed in only males, and 10 DEGs were differentially expressed in only females (Fig. 5F, left). When -log(*p*-value) of the genes encoding for mitochondrial subunits were plotted in a line graph, some distinct expressions between the males and females were observed, which confirmed that some genes were significantly differentially expressed in only one of the sexes, indicative of quantitative rather than qualitative differences between the sexes for this process (Fig. 5F, right).

### AKT signaling is regulated in both sexes, but with a greater magnitude in females

**Fig. 6:**
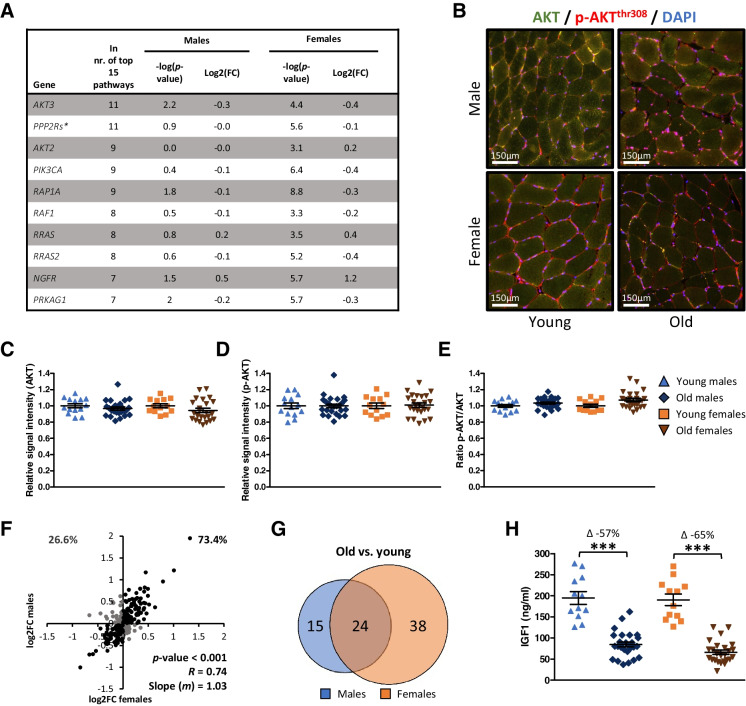
Effects of aging on AKT signaling in males and females. (**A**) Top 10 genes ranked on the number of times a gene is a part of one of the top 15 differentially expressed pathways of female-specific DEGs. *PPP2Rs are average values of multiple PPP2R subunits. (**B**) Representative pictures of AKT and p-AKT^thr308^ immunofluorescence staining (20× magnification). (**C**) Relative AKT and (**D**) p-AKT^thr308^ protein content in young males (*n* = 13), old males (*n* = 25), young females (*n* = 13), and old females (*n* = 25). (**E**) Ratio of p-AKT^thr308^ to AKT signal. (**F**) Correlation plot of log2FC values of genes involved in AKT signaling. Genes were selected using the GO term “protein kinase B signaling” (GO:0043491). Fold change values of old vs. young males (*x*-axis) and old vs. young females (*y*-axis) were used. *P*, *R*, and *m* values are based on all data points in this plot. Percentages in the upper corners of this correlation plot represent the percentage of DEGs regulated in the opposite (Q1 and Q4) or the same (Q2 and Q3) directions in males and females. (**G**) Venn diagram of the number of genes involved in AKT signaling that are regulated in both or only one of the sexes (left). Line graph of *p*-values of these genes ranked on their average -log(*p*-value) in males and females. (**H**) IGF1 serum concentrations in young males (*n* = 12), old males (*n* = 27), young females (*n* = 12), and old females (*n* = 25)

Pathway analysis of female-specific DEGs revealed mainly pathways involved in growth signaling. *AKT3* and *AKT2* were part of 11 and 9 out of 15 pathways, respectively, revealing that AKT signaling played an important role in growth signaling pathways that were affected by aging in females (Fig. 6A). To measure AKT activity, we performed a double immunofluorescence staining of AKT and p-AKT^(thr308)^ (Fig. 6B), but no differences between old and young participants were found in both sexes (Fig. 6C–E). We then selected genes involved in AKT signaling, and fold change values of old vs. young groups of both sexes were plotted (Fig. 6D). A significant correlation was revealed (*p* < 0.001, *R* = 0.77), and 73% of these genes were regulated in the same direction in females and males. Fold change values were highly similar in males and females, as indicated by the slope-value *m* of 1.03, although more genes related to AKT signaling were differentially expressed in females compared to males (Fig. 6E). All in all, these data suggest that cell growth signaling is negatively regulated in both sexes during aging, but quantitatively to a stronger degree in females, since more DEGs for cell growth signaling were found in females compared to males. AKT signaling is part of many pathways. To explore possible upstream regulation, we noticed “IGF1 signaling” being one of the most prominently differently expressed pathways in females (Fig. 4F). A significant decrease in serum IGF1 levels was seen in old females (66.5 ± 5.3 ng/ml) compared to young females (190.6 ± 13.7 ng/ml, *p* < 0.001), and in old males (84.7 ± 5.8 ng/ml) compared to young males (195.1 ± 14.9 ng/ml, *p* < 0.001, Fig. 6F).

### Findings in RNA-seq data of the current study are confirmed by external GEO datasets

In order to substantiate our findings, we resorted to publicly available external data, namely GEO datasets of studies with a similar study population and with muscle biopsies taken from the *vastus lateralis* muscle as well. We found three datasets that fulfilled the selection criteria, but the authors did not compare old (≥65 years) vs. young (18–30 years) males and females separately themselves [29–32], which we did here.

**Fig. 7:**
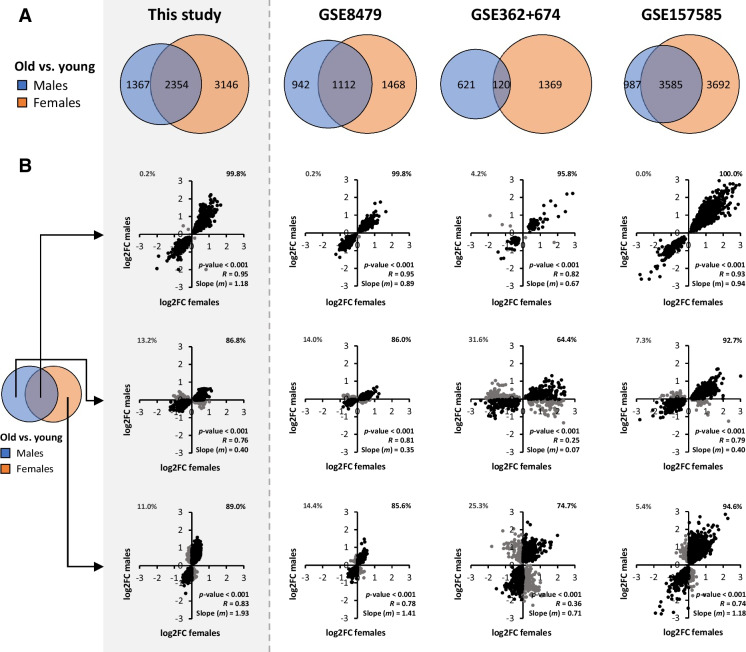
Bioinformatic analysis of transcriptome data of three external GEO datasets to validate previous results. (**A**) Venn diagrams of the number of DEGs in old vs. young males and females for each study separately. (**B**) Correlation plots of log2FC values of old vs. young males and females to assess sex differences in the direction of regulation (up or down) of shared or sex-specific DEGs. In all correlation plots, *p*, *R*, and *m* values are based on all data points in a plot. Log2FC values of old vs. young males are plotted on the *x*-axis, and log2FC values of old vs. young females are plotted on the *y*-axis. Percentages in the upper corners of this correlation plot represent the percentage of DEGs regulated in the opposite (Q1 and Q4) or the same (Q2 and Q3) directions in males and females. The upper row of correlation plots displays log2FC values of shared DEGs, the middle row displays log2FC values of male-specific DEGs, and the lower row displays log2FC values of female-specific DEGs for each dataset separately

In the current study, 48% more genes were significantly differentially regulated in females compared to males during the aging of *vastus lateralis* muscle (5500 vs. 3721 DEGs, Fig. 7A). Similarly, in other studies, 26%, 100%, and 59% more genes were regulated in females compared to males (GSE8479, GSE362+674, and GSE157585, respectively, Fig. 7A). This confirmed our finding that in old vs. young females, more genes are differentially expressed than in old vs. young males. To investigate whether, in these external studies, sex differences were also primarily found in the magnitude and not in the direction of differential expression, we performed correlation analyses of DEGs that were sex specific or were shared by the two sexes (Fig. 7B).

Genes that were regulated in both sexes were almost always regulated in the same direction in the current study (99.8%, *R* = 0.95, *m* = 1.18), and also in all of the external datasets (99.8%, *R* = 0.95 in GSE8479; 95.8%, *R* = 0.82 in GSE362+674 and 100%, *R* = 0.93 in GSE157585, Fig. 7B). Fold change values in males and females were highly similar, since slope-values *m* were close to 1 in these studies as well, which underpins that some features of muscle aging are conserved across males and females.

Furthermore, for male-specific DEGs, we found that 86.8% (*R* = 0.76, *m* = 0.40) were regulated in the same direction in both sexes. This observation was reproduced in GSE84797 (86.0%, *R* = 0.81, *m* = 0.35) and GSE157585 (92.7%, *R* = 0.79, *m* = 0.40). In GSE362+674, a lower percentage of overlap in the direction of regulation was found (64.4%, *R* = 0.25, *m* = 0.07) for male-specific DEGs.

The direction of regulation of female-specific DEGs was also highly similar in the current study (89.0%, *R* = 0.83, *m* = 1.93). This was also reproduced in GSE84797 (85.6%, *R* = 0.81, *m* = 1.41), GSE362+674 (74.7%, *R* = 0.36, *m* = 0.71), and GSE157585 (94.6%, *R* = 0.74, *m* = 1.18). Together, these external studies confirm our finding that sex-specific effects of aging on the transcriptome of *vastus lateralis* muscle are primarily found in the magnitude of regulation and not in the direction of regulation.

## Discussion

The aim of this study was to investigate whether sex-specific muscle-aging mechanisms could be identified. Due to the careful matching of male and female participants and the homogeneity of the groups, we were able to compare the differences found in old vs. young groups of either sex. We found that the gene expression patterns that discriminate old from young muscle tissue were highly similar in males and females, although there were some differences. We found that in old vs. young females, more genes were differentially expressed than in old vs. young males, and that the majority of all DEGs were significantly differentially expressed in only one of the sexes. However, we also found that the direction of regulation of these sex-specific DEGs was in fact highly similar in both sexes. This revealed that the same processes were associated with muscle aging in males and females, but that the magnitude of the differential gene expression was sex specific. This is an important observation, as this suggests that sex-specific events that occur during aging (e.g., menopause) do not necessarily introduce a muscle aging-related process that is qualitatively unique for one of the sexes. To our knowledge, the results of the current study are unique compared to other studies, as we chose to focus our analysis on the differences between old vs. young participants of both sexes. Consequently, our results reflect the sex differences in the aging trajectory per se.

With regard to sex differences in aging-related changes in energy metabolism, we have shown on both mRNA and protein level that sex differences in the aging-related loss of OXPHOS subunits were very small (Fig. 5). We were able to confirm this finding on mRNA level using the external, publicly available datasets as well (Supplementary Fig. 3A). This suggests that loss of OXPHOS-associated energy expenditure throughout the humane life course is similar in males and females. This finding is fully in line with the pertinent observation that after adjusting for body size, similar changes in energy expenditure throughout the life course occur in males and females [34]. On the other hand, our findings challenge the observations of several other studies regarding sex differences in aging-related loss of mitochondrial content. One study showed that aging-related loss of intermyofibrillar mitochondrial area occurred in the *vastus lateralis* muscle of old vs. young females, but not in old vs. young males [18], which suggested that loss of mitochondrial content is female specific. This finding is challenged by our data, and the differences in our findings could possibly be explained by the low number of included participants in the other study using electron microscopy. Our findings also challenge the findings of a meta-analysis, in which it was found that muscle oxidative capacity was higher in old vs. young males, but lower in old vs. young females [35]. However, the populations used in the meta-analysis were very heterogeneous (e.g., trained and sedentary participants were included, and both young and adult participants were used as control groups), and the exact comparisons that were done were poorly described. Furthermore, it could be argued that mRNA levels encoding OXPHOS subunits do not necessarily translate into oxidative function; however, this is unlikely in the light of previously reported significant correlations between OXPHOS subunit levels and *in vivo* mitochondrial capacity in old and young male participants [22].

To our knowledge, only one study also compared old vs. young groups of both sexes using transcriptomics, but used biopsies taken from the *biceps branchii* muscle [36]. In the *biceps branchii*, a stronger decrease in the expression of genes involved in mitochondrial structure and function was found in old vs. young females compared to old vs. young males. This is different from our findings, as we found a highly similar reduction of expression of genes involved in OXPHOS in both sexes (Fig. 5E). In addition, in the *biceps branchii* muscle, the most differentially expressed processes in females were reported to be associated to the extracellular matrix and immune function [36], while we observed that the most prominent female-specific pathways were involved in cell growth signaling. Perhaps, these different findings can be explained by differences in aging trajectories in muscles of arms and legs [37], although this has not been studied in detail yet and a number of other differences could also provide an explanation, such as differences in age of the participants or the matching of males and females.

We found more changes in the IGF1/PI3K/AKT signaling axis in old vs. young females compared to old vs. young males, and we also found this in two of the three external GEO datasets (Supplementary Fig. 3B). Notably, we have found no differences in AKT or p-AKT^308^ levels in old vs. young participants of both sexes. Possibly, aging-related differences in AKT phosphorylation levels become apparent only after an anabolic stimulus (e.g., resistance exercise training) [38, 39], and not in basic, resting condition after an overnight fast as was the case in the current study. Furthermore, IGF1 is an important upstream regulator of PI3K and AKT and is implicated in aging [40]. Associations between circulating IGF1 and skeletal muscle mass in the elderly have been reported [41]. We found a significant decrease in serum concentrations of IGF1 in old vs. young participants of both sexes. This shows that aging-related loss of circulating IGF1 is not unique for one of the sexes, although changes in its downstream effects on the skeletal muscle transcriptome were of higher amplitude in old vs. young females compared to old vs. young males. This finding is in line with the findings of a cross-sectional trial in which lower serum IGF1 levels were found to be associated with the prevalence of sarcopenia in females, but not in males [21]. The observed sexual dimorphism here could possibly be explained by other intramuscular aging-related changes related to IGF1 signaling, which would then be of higher magnitude in females compared to males.

Furthermore, interestingly, we found an aging-related shift toward a type 1 (slow) myofiber dominant composition in males and not in females. Similar to other studies [42, 43], we observed a higher proportion of type 1 myofibers in young females (52.7%) compared to young males (46.4%), but importantly, our findings now suggest that during aging, only in males, a shift toward a type 1 (slow) myofiber dominant composition occurs. Perhaps, the proportion of type 1 myofibers was not increased in old vs. young females, because females already possess more type 1 myofibers when they are young. Consequently, females could be less susceptible to the aging-related reduction of type 2 myofibers. This is a unique and novel finding, which has to be reproduced and studied in greater detail in future studies.

These results have implications for sarcopenia as well since muscle aging is an important driver of sarcopenia [1]. We showed that processes associated with muscle aging, when comparing 80 to 20 years old participants, are not necessarily sex specific. This is especially the case for pathways that were found using shared DEGs as input, including sirtuin signaling, protein ubiquitination, and mitochondrial respiration (Fig. 4B,C). Based on these results, it can be hypothesized that mechanisms associated with sarcopenia are also highly similar in males and females, but their magnitude may be sex dependent. This may shed light on the mixed results that are reported by multiple studies, as some conclude that sarcopenia is more prevalent in males [16, 44], while others conclude the contrary [45, 46]. However, the inconsistency in these results can possibly also be explained by the fact that the cut-off values used to diagnose sarcopenia can highly influence the diagnostic outcome, especially in female older adults [17]. Furthermore, our data show that the processes associated with muscle aging are not necessarily sex specific; however, we did not study the onset of the age-related gene expression changes. Since the timing of the muscle aging-related processes could be sex specific [47], it would be of interest to include additional age groups in future studies. In addition, future research could also study potential sex differences in intramuscular changes associated with frailty since sex differences in the prevalence and functional manifestation of frailty have consistently been reported in both humans and pre-clinical models [48].

A limitation of our study is its observational study design. By using this study design, it is difficult to exclude the presence of potential confounders, such as differences in the lifestyle of young vs. old groups. We did take this into account by matching old and young groups of either sex as much as possible and by excluding many aging-related diseases. In addition, by comparing our results with those of external datasets, we showed that our results are reproducible, rendering the presence of significant confounders unlikely. Future studies could match old and young participants for physical activity as well. Another limitation of this study is that the results found in the *vastus lateralis* muscle may possibly not be extrapolated toward other muscle groups, as aging trajectories appear to be muscle specific. Aging trajectories are likely to be different in muscles of lower and upper extremities [37], and likely even also in muscles within the same extremity [23], due to, e.g., different myofiber type compositions, changes in posture [49], or age-related changes in neuromuscular activation during physical activity [50].

We identified molecular processes associated with the aging trajectory of the *vastus lateralis* muscle and concluded that similar processes are associated with skeletal muscle aging of males and females, but the magnitude of differential expression of these processes in old vs. young participants is sex specific. Loss of OXPHOS subunits is similar in male and female aging, while changes in growth signaling, via AKT signaling, seemed stronger in female aging. Our results show the importance of discriminating between pre-existing sex differences in young groups and sex differences in the aging trajectory itself, as we found that the latter was qualitatively not different between the two sexes. Our findings provide novel insights into muscle aging that create leads for further studies and ultimately may help the prevention of sarcopenia.

### Supplementary information

ESM 1: Supplementary Fig. 1 Flowchart describing the process of selecting viable external GEO datasets. Supplementary Fig. 2 Myofiber size distribution based on their minimal Feret’s diameter. (A) Type 1 myofibers and (B) type 2 myofibers. Supplementary Fig. 3 Upstream regulator analysis. (A) top 15 male and (B) female upstream regulators. Supplementary Fig. 4 Bioinformatic analysis from external GEO-studies on genes involved in top male differentially expressed pathways (OXPHOS) or top female differentially expressed pathways (AKT signaling). Genes encoding for OXPHOS subunits were selected using the MitoCarta 3.0 inventory [33], and genes involved in AKT signaling were selected using the GO term “protein kinase B signaling” GO:0043491. (A) Venn-diagrams of number of male or female DEGs, and correlations graphs of old vs. young male and female log2FC values of OXPHOS genes. (B) Venn-diagrams of number of male or female DEGs, and correlations graphs of old vs. young male and female log2FC values of genes involved in AKT signaling.
