# Supplementary figures and images for: Sex differences in skeletal muscle-aging trajectory: same processes, but with a different ranking
Source: GeroScience. 2023 Feb 23;45(4):2367–86. doi: 10.1007/s11357-023-00750-4 (PMC10651666; doi:10.1007/s11357-023-00750-4)

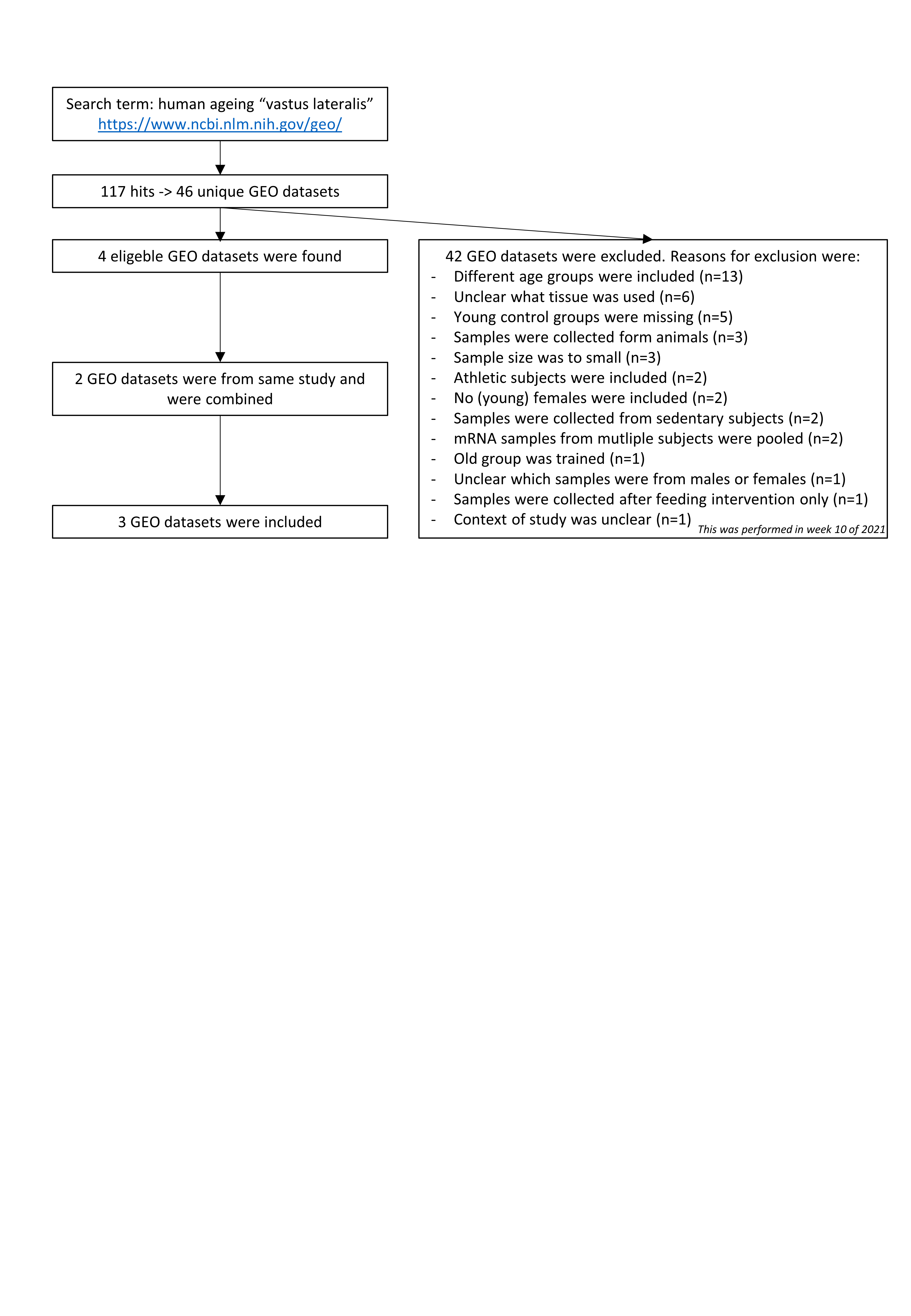

Supplement: Supplementary file 1 — Supplementary Fig. 1 Flowchart describing the process of selecting viable external GEO datasets. Supplementary Fig. 2 Myofiber size distribution based on their minimal Feret’s diameter. (A) Type 1 myofibers and (B) type 2 myofibers. Supplementary Fig. 3 Upstream regulator analysis. (A) top 15 male and (B) female upstream regulators. Supplementary Fig. 4 Bioinformatic analysis from external GEO-studies on genes involved in top male differentially expressed pathways (OXPHOS) or top female differentially expressed pathways (AKT signaling). Genes encoding for OXPHOS subunits were selected using the MitoCarta 3.0 inventory [33], and genes involved in AKT signaling were selected using the GO term “protein kinase B signaling” GO:0043491. (A) Venn-diagrams of number of male or female DEGs, and correlations graphs of old vs. young male and female log2FC values of OXPHOS genes. (B) Venn-diagrams of number of male or female DEGs, and correlations graphs of old vs. young male and female log2FC values of genes involved in AKT signaling. [file 11357_2023_750_MOESM1_ESM.zip › Suppl_Fig_1.tif]

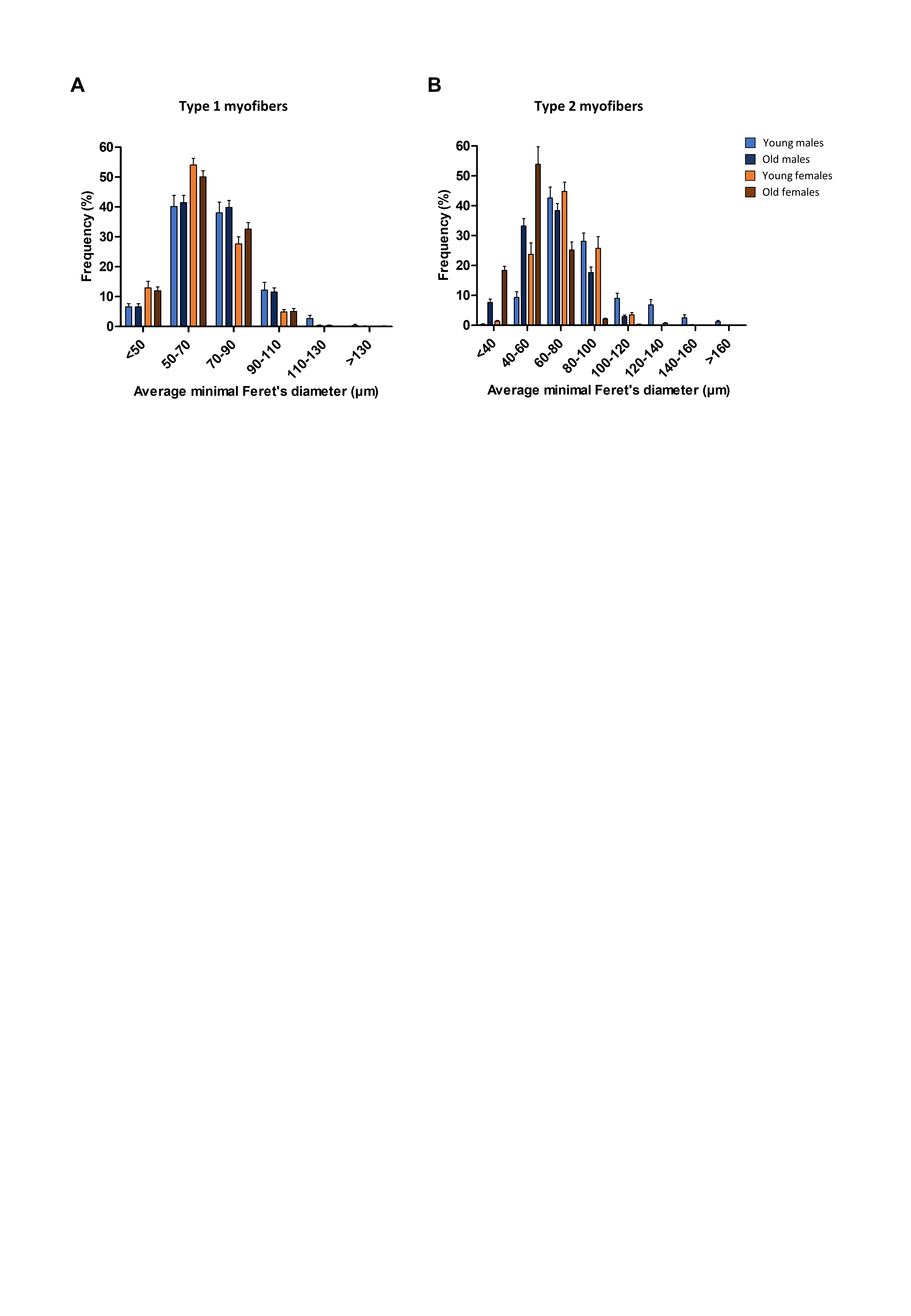

Supplement: Supplementary file 1 — Supplementary Fig. 1 Flowchart describing the process of selecting viable external GEO datasets. Supplementary Fig. 2 Myofiber size distribution based on their minimal Feret’s diameter. (A) Type 1 myofibers and (B) type 2 myofibers. Supplementary Fig. 3 Upstream regulator analysis. (A) top 15 male and (B) female upstream regulators. Supplementary Fig. 4 Bioinformatic analysis from external GEO-studies on genes involved in top male differentially expressed pathways (OXPHOS) or top female differentially expressed pathways (AKT signaling). Genes encoding for OXPHOS subunits were selected using the MitoCarta 3.0 inventory [33], and genes involved in AKT signaling were selected using the GO term “protein kinase B signaling” GO:0043491. (A) Venn-diagrams of number of male or female DEGs, and correlations graphs of old vs. young male and female log2FC values of OXPHOS genes. (B) Venn-diagrams of number of male or female DEGs, and correlations graphs of old vs. young male and female log2FC values of genes involved in AKT signaling. [file 11357_2023_750_MOESM1_ESM.zip › Suppl_Fig_2.tif]

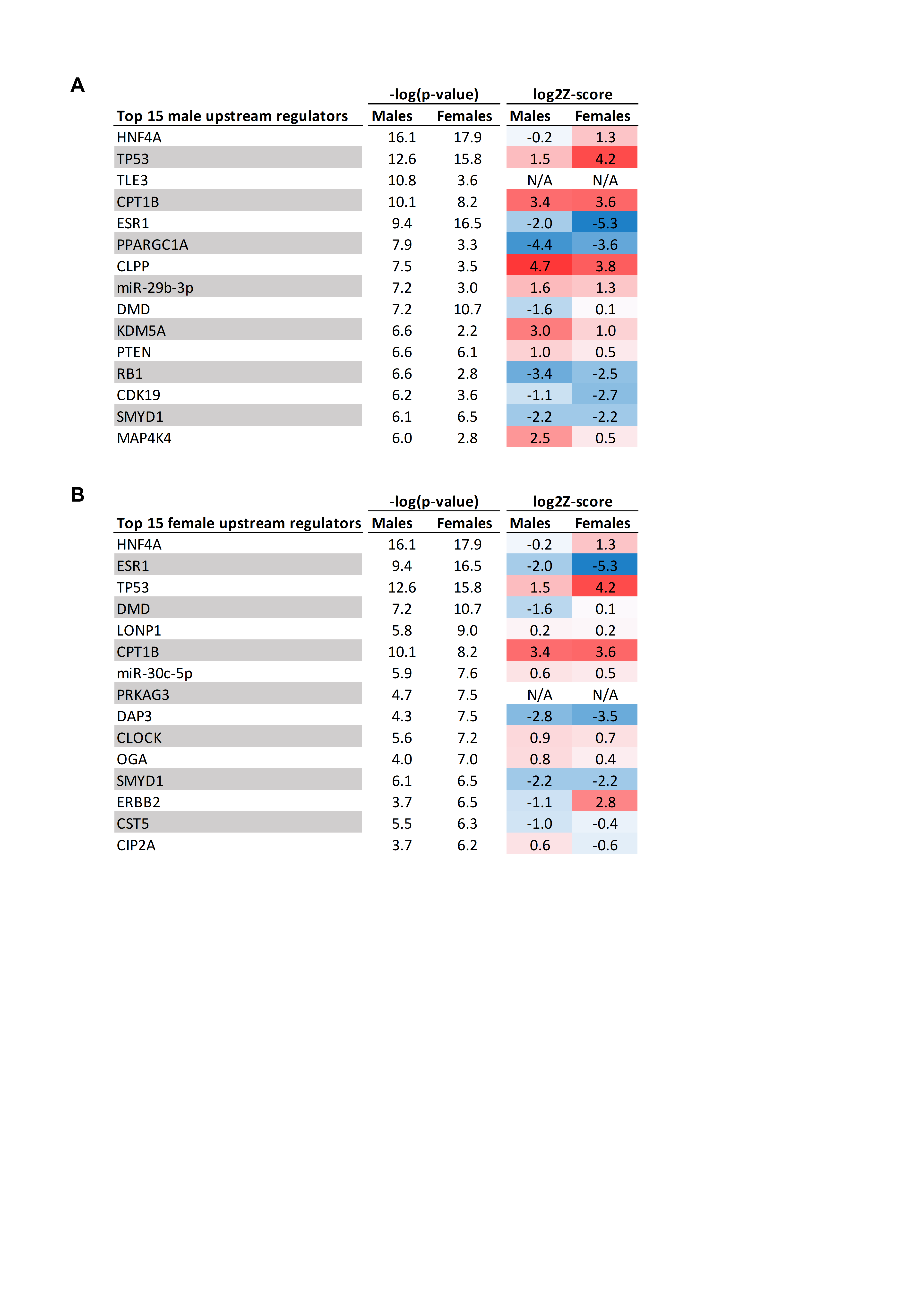

Supplement: Supplementary file 1 — Supplementary Fig. 1 Flowchart describing the process of selecting viable external GEO datasets. Supplementary Fig. 2 Myofiber size distribution based on their minimal Feret’s diameter. (A) Type 1 myofibers and (B) type 2 myofibers. Supplementary Fig. 3 Upstream regulator analysis. (A) top 15 male and (B) female upstream regulators. Supplementary Fig. 4 Bioinformatic analysis from external GEO-studies on genes involved in top male differentially expressed pathways (OXPHOS) or top female differentially expressed pathways (AKT signaling). Genes encoding for OXPHOS subunits were selected using the MitoCarta 3.0 inventory [33], and genes involved in AKT signaling were selected using the GO term “protein kinase B signaling” GO:0043491. (A) Venn-diagrams of number of male or female DEGs, and correlations graphs of old vs. young male and female log2FC values of OXPHOS genes. (B) Venn-diagrams of number of male or female DEGs, and correlations graphs of old vs. young male and female log2FC values of genes involved in AKT signaling. [file 11357_2023_750_MOESM1_ESM.zip › Suppl_Fig_3.tif]

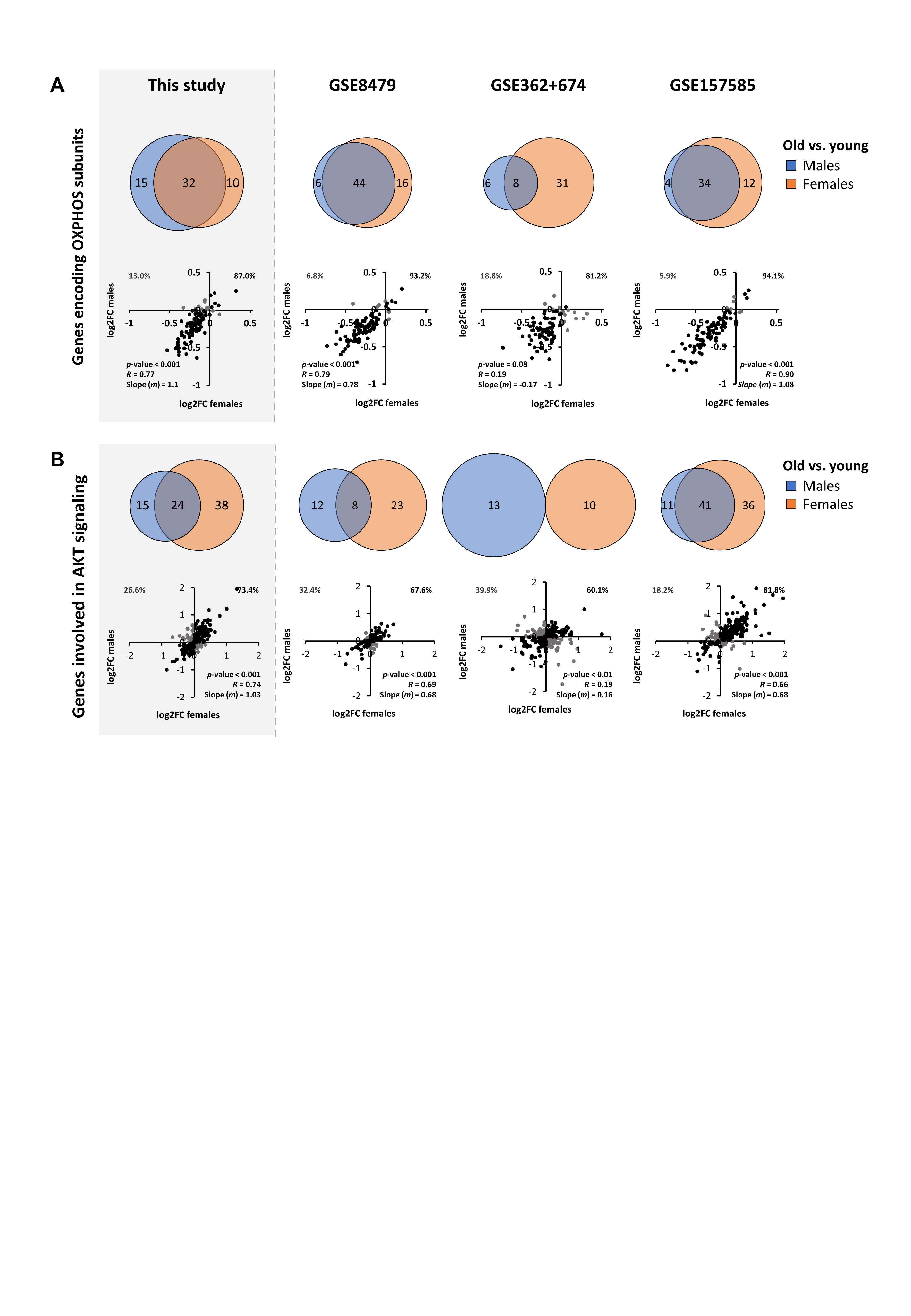

Supplement: Supplementary file 1 — Supplementary Fig. 1 Flowchart describing the process of selecting viable external GEO datasets. Supplementary Fig. 2 Myofiber size distribution based on their minimal Feret’s diameter. (A) Type 1 myofibers and (B) type 2 myofibers. Supplementary Fig. 3 Upstream regulator analysis. (A) top 15 male and (B) female upstream regulators. Supplementary Fig. 4 Bioinformatic analysis from external GEO-studies on genes involved in top male differentially expressed pathways (OXPHOS) or top female differentially expressed pathways (AKT signaling). Genes encoding for OXPHOS subunits were selected using the MitoCarta 3.0 inventory [33], and genes involved in AKT signaling were selected using the GO term “protein kinase B signaling” GO:0043491. (A) Venn-diagrams of number of male or female DEGs, and correlations graphs of old vs. young male and female log2FC values of OXPHOS genes. (B) Venn-diagrams of number of male or female DEGs, and correlations graphs of old vs. young male and female log2FC values of genes involved in AKT signaling. [file 11357_2023_750_MOESM1_ESM.zip › Suppl_Fig_4.tif]
